# Does Psychological Resilience Affect Sexual Satisfaction in Women with Polycystic Ovary Syndrome? A Cross-Sectional Observational Study

**DOI:** 10.1192/j.eurpsy.2025.1223

**Published:** 2025-08-26

**Authors:** A. Bicer, A. C. Kahve, D. G. Mert, B. Elmas, E. Göka

**Affiliations:** 1 Psychiatry, Health Sciences University Ankara City Hospital; 2 Psychiatry, Gazi University; 3 Obstetrics and Gynecology, Health Sciences University Ankara City Hospital, Ankara, Türkiye

## Abstract

**Introduction:**

Polycystic Ovary Syndrome (PCOS) is a common endocrine disorder in women of reproductive age, characterized by hyperandrogenism, oligomenorrhea/amenorrhea, and polycystic ovary morphology. PCOS can negatively impact sexuality due to physical issues like hyperandrogenism, metabolic syndrome, and infertility, as well as the psychological burden of the disease and associated mental health issues. Psychological resilience, defined as the ability to cope with stress and recover, is known to aid in managing physical illnesses and improve treatment outcomes. Although psychological resilience and sexuality are individually studied in relation to physical and psychological problems, research on their relationship, especially in PCOS, is limited.

**Objectives:**

This study aimed to explore the relationship between psychological resilience and sexual satisfaction in women with PCOS, comparing it with a healthy control group, and to identify factors affecting sexual satisfaction in PCOS patients.

**Methods:**

70 women aged 18-40 with a PCOS diagnosis and 69 healthy controls matched for age and education participated in this study. Participants underwent a psychiatric interview according to the SCID-5 and completed the Sociodemographic Data Form, Psychological Resilience Scale for Adults (RSA), Hospital Anxiety and Depression Scale (HADS), Golombok Rust Inventory of Sexual Satisfaction (GRISS), and Arizona Sexual Experiences Scale (ASEX). Blood tests requested by the gynecologist were also documented for the PCOS group.

**Results:**

Women with PCOS exhibited lower psychological resilience than healthy controls, particularly in self perception area as shown in **Table 1**. They also reported lower sexual satisfaction with partners. No significant difference was found in masturbation frequency between the groups, nor was masturbation related to sexual satisfaction in either group. However, increased psychological resilience was associated with higher sexual satisfaction in both groups, with future perception area on the RSA significantly impacting sexual satisfaction. Examination of the relationship between RSA scores and GRISS and ASEX scores in women with PCOS is presented in **Table 2** and **Table 3**.

**Image 1:**

**Figure:**
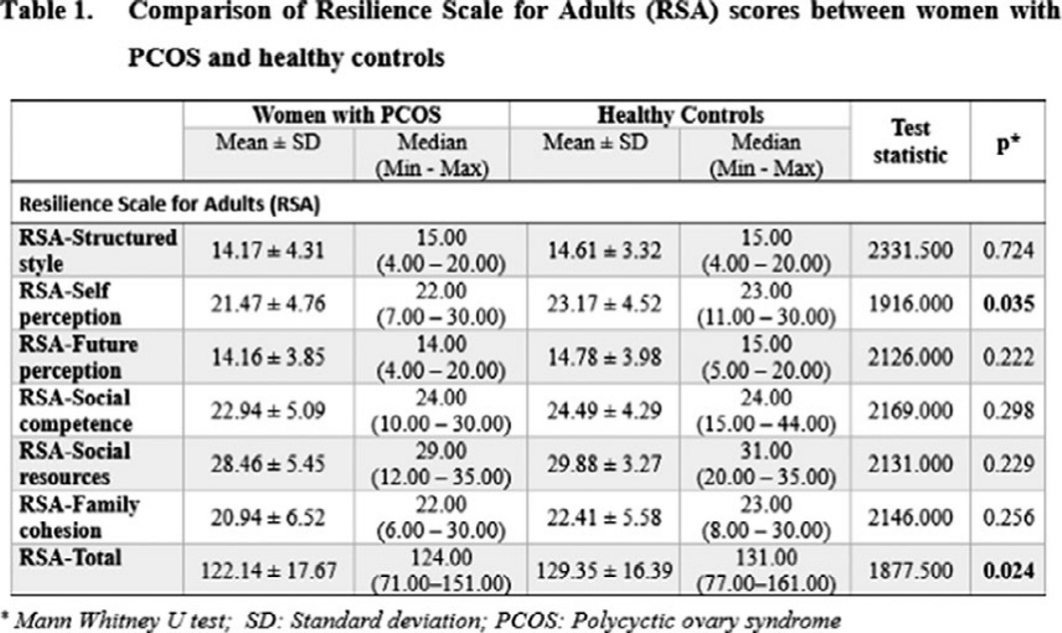

**Image 2:**

**Figure:**
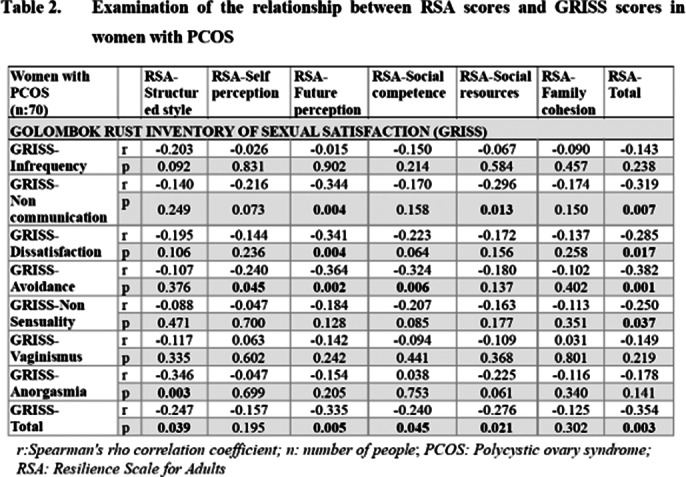

**Image 3:**

**Figure:**
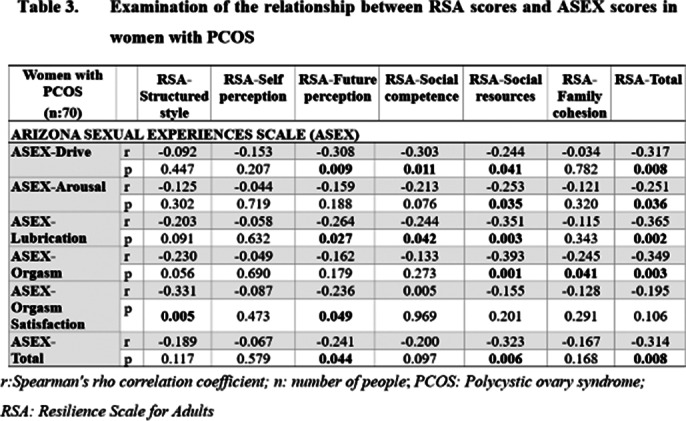

**Conclusions:**

Psychological resilience, especially the future perception area, may play an influential role in sexual satisfaction. In light of these findings, it can be suggested that the treatment of PCOS should involve a multidisciplinary approach that includes mental health professionals. It is important to evaluate the sexual life of these women during follow-ups, provide psychoeducation, and adopt a hopeful approach. Considering factors that influence psychological resilience, interventions aimed at reducing risk factors and increasing protective factors could be anticipated to be important tools in preventing and treating sexual problems.

**Disclosure of Interest:**

None Declared

